# Supplementary material for: Chikungunya virus (CHIKV) seroprevalence in the South Pacific populations of the Cook Islands and Vanuatu with associated environmental and social factors
Source: PLoS Negl Trop Dis. 2022 Nov 28;16(11):e0010626. doi: 10.1371/journal.pntd.0010626 (PMC9731434; doi:10.1371/journal.pntd.0010626)
Supplement: Attachment — Submitted filename: Letter to reviewers_with answers 21.08.2022.pdf [file pntd.0010626.s002.pdf]

Dear reviewers,

first, we would like to thank you for careful reviewing. In the revised version we have included all your ideas and suggestions. To facilitate your work, we have not added a letter containing a detailed list, but we have added our responses directly in your text (written in blue).

There is one remaining question concerning one of Reviewer #1's comments in the results-section (this one is written in red). Maybe you could help us understanding what is meant here so we can adapt the manuscript as desired.

Please find attached two versions of the revised manuscript: one with tracked changes and a clean version.

Further, we will upload our figure files via the PACE tool as well as our data as supporting information.

Thank you again for the fast review and please don't hesitate to contact us in case there should be anything missing.

Sincerely,

Thomas Kuepper

Charlotte Saretzki

Answers to Reviewers Responses

Key Review Criteria Required for Acceptance?
As you describe the new analyses required for acceptance, please consider the following:

Methods
-Are the objectives of the study clearly articulated with a clear testable hypothesis stated?
-Is the study design appropriate to address the stated objectives?
-Is the population clearly described and appropriate for the hypothesis being tested?
-Is the sample size sufficient to ensure adequate power to address the hypothesis being tested?
-Were correct statistical analysis used to support conclusions?
-Are there concerns about ethical or regulatory requirements being met?

Reviewer #1:

The objectives are pretty well described, however I would suggest as per PLOSNTD 'suggestions' to:
a) relocate sentences 105-109 to Methods section,

- Sentences were relocated as desired

b) amend formulation > To assess the seroprevalence of CHIKV a study has been conducted in the local / (or [apparently not as expatriates were not excluded]? — age limitations?) population of the Cook Islands, which had been affected by an outbreak in 2014/15 and Vanuatu, which had never reported such an outbreak. In both study setting specific environmental variables were assessed … (specify why?).

- formulation is amended as desired
- we added the term "permanent inhabitants" to describe the study population more accurately
- for sample collections there was no age limitation
- we added a statement why environmental variables were assessed (influence on vector spread)

c) consider to add a sentence on why you selected these two islands except for the yes/no reason. There would have been many other options.

- we added a statement, why the Cook Islands and Vanuatu were chosen (comparison of a Polynesian to a Melanesian country)

With such additional information the objective will be crystal-clear, the population clearly described. The sample size is sufficient for the simple statistical analysis needed here, except that on Vanuatu the sample size is too small apparently lacking cases in small children to show that seroprevalence has recently been acquired. No concerns about ethics or regulatory issues.

DETAILS:
114-166 I presume there is only one hospital lab each in Espiritu Santo, Rarotonga and Aitutaki. If so be more precise, if you selected single labs, explain why.

- We clarified that there is only one hospital lab on each island

119 Do not start sentence with a number or write 'Fourhundred'..'.

- We changed the start of the sentence

Reviewer #2:

objectives clear, study design appropriate. population clearly indicated, sample size sufficient, statistical methods ok, no ethical concerns

----

Results
-Does the analysis presented match the analysis plan?
-Are the results clearly and completely presented?
-Are the figures (Tables, Images) of sufficient quality for clarity?

Reviewer #1:

Essentially well described. Particularly appreciated that the results in the text and figures / tables were complimentary and not repetitive.

DETAIL:
206 Why was age group 0-9y excluded? No data? Would have been relevant, see above.

- as described in the Method section, the age group 0-9 was excluded from analysis due to low case numbers (in the Cook Islands collective there were only 2 patients in this age group)

213 25.0?

- We do not fully understand this comment

220-226 As the Cook Island outbreak has not been part of the study per se I would rather move that to the Discussion (or Introduction).

- the section was moved to *Discussion*

Reviewer #2:

Analysis matches the plan, results clear, tables to be slightly modified (as indicated)

- Table was modified as suggested

Conclusions
-Are the conclusions supported by the data presented?
-Are the limitations of analysis clearly described?
-Do the authors discuss how these data can be helpful to advance our understanding of the topic under study?
-Is public health relevance addressed?

Reviewer #1:

Essentially agree with the conclusions presented from 293. Suggest to add the word 'international' on line 302 to more clearly differ from local mobility.

- We added the word “international”

Limitations are clearly described, possibly an addition should be made relating to the pediatric population.

- We added a sentence emphasizing that the age group 0-9 years was excluded from some parts of the analysis.

- We further added that information concerning the role of Ae. hebrideus in CHIKV transmission are missing

Suggest to add a final conclusive sentence on the public health relevance and the need of further assessment in the SPR.

- We shifted line 304-307 to the end of the Discussion section and added a sentence pointing out the need for further studies

Reviewer #2:

conclusions clear. limitations correctly indicated, contributions indicated, PH relevance addressed

----

Editorial and Data Presentation Modifications?
Use this section for editorial suggestions as well as relatively minor modifications of existing data that would enhance clarity. If the only modifications needed are minor and/or editorial, you may wish to recommend “Minor Revision” or “Accept”.

Reviewer #1:

Suggest to modify the title: Chikungunya virus seroprevalence in the South Pacific populations of the Cook Islands and Vanuatu with associated environmental and social factors. > delete the second sentence which does not offer much additional information.

- We deleted the second sentence

While the English is pretty good, sometimes the wording is a bit clumsy (possibly from German background) > suggest to have a native British or American speaker with epidemiological knowledge check the manuscript.

- The manuscript was checked by a native British speaker with epidemiological knowledge and several linguistic corrections were carried out

PLOS NTD does not publish any titles like 'full professor', 'MD' > delete. Anyhow you have been inconsistent. See instructions for authors.

- All titles were deleted

59. Suggest 'Arboviral' instead of 'mosquito-borne viral'

- The term was changed

95. Are subregions essential for this paper? Rather not.

- Yes, they are important as there are major differences in terms of population mobility as described below

98. Are natural disasters essential for this seroprevalence study?

- Yes, they are important as they are an important characteristic of the SPR, add to the challenges health systems must face, and make populations more vulnerable to infectious diseases

Suggest you review the manuscript only keeping the essential elements.

50, 107, 293: no need to perseverate on 'first study' in these two islands. After all there was a seroprevalence of about 3% in French Polynesia possibly before 2014.

- The term was deleted in lines 107 and 293.

DISCUSSION:

228-229 Initial sentence rather trivial > suggest to delete and start: Subsequent to the 2014/15 CHIKV outbreak, 30% showed evidence ...

- The first sentence was moved to the end of the paragraph and the discussion starts as suggested

231 Suggest: ... differences between the two Cook Islands [the names will be specified in 234]

- island names in brackets were deleted

Reviewer #2:

COMMENTS IN DETAIL:

ABSTRACT:
BACKGROUND:
P(age) 3 / L(ine) 35: to my knowledge there is no local transmission in Australia as by today

- You're right; we changed the sentence

AUTHOR SUMMARY
P4 / L60: this is not really true; e.g. WNV circulates in the USA and in Europe too; maybe better: „as most of them predominantly circulate in (sub-)tropical regions…"

- The sentence was changed as suggested

INTRODUCTION
P 5 / L 78: „global burden of disease" instead of „global disease burden"

- The term was changed as suggested

… / L79: are yellow fever virus, japanese encephalitis virus, west nile virus, tick borne encephalitis virus not important? Maybe better: amongst all arboviruses the CHIKV adds …

- The sentence was changed. We added that there was little interest in CHIKV for a long time

… / L86: well it was not the extent only but also the speed (e.g. Dominican Republic: roughly 540.000 cases within 15 months) which might also be due to the aggressive biting behaviour of Aedes ssp. in contrast e.g. to Culex ssp.

- We added "rapid dissemination"

… / L88: [6,7,8] instead of [6],[7],[8]

- Changed as suggested

… / L90: [8,9,10] instead of [8],[9],[10]

- Changed as suggested

P 6 / L 102: [9,10,13,14,15,16,17] instead of [9],[10],[13],[14],[15],[16],[17]
„local burden of disease" instead of „local disease burden"

- Changed as suggested

… / L 104: [9,10] instead of [9],[10]

- Changed as suggested

MATERIAL AND METHODS

P 7 / L 114: I wonder whether it would be better to place the comment on the votum by the ethic committee (L 124-127) at the beginning of this chapter

- The votum was shifted further forward.

RESULTS

P 10 / Table: you indicate „warmest month“ and „coldest month“ with the temperature in brackets and in the colums you indicate the temperature with the month in brackets and with the precipitation you indicate the mm in brackets whereas you show the month in brackets which is not even indicated in the first column; if you use […] for description oft he value you could indicate : temperature [oC] (warmest month). Temperature [oC] (coldest month), highest precipitation [mm] (month), lowest precipitation [mm] (month)

- Table was modified as suggested

misspelling in Ae. hebrideus

- Misspelling was corrected

P12 / L190: „specimens“ instead of „specimen“ (..L191 idem)

- Corrected as suggested

DISCUSSION

P15 / L240: „real burden of disease“ instead of „real disease burden“

- Corrected as suggested

…/L 241: [2,13] instead of [2],[13]

- Changed as suggested

P16 / L261: [32,40,41] instead of [32],[40],[41]

- Changed as suggested

…/L277: [21,46,47,48,49,50] instead of [21],[46],[47],[48],[49],[50]

- Changed as suggested

P17 / L280: [12,15] instead of [12],[15]

- Changed as suggested

P18 / L316: [52,53] instead of [52],[53]

- Changed as suggested

REFERENCES
P23 / L 419: „cyclone“ instead of „cyclon“

- Corrected as suggested
- Due to text modifications references 38 and 39 had to be switched

  ----

Summary and General Comments</br></br>
Use this section to provide overall comments, discuss strengths/weaknesses of the study, novelty, significance, general execution and scholarship. You may also include additional comments for the author, including concerns about dual publication, research ethics, or publication ethics. If requesting major revision, please articulate the new experiments that are needed.

Reviewer #1: Well conducted study which adds to the perception of CHIKV epidemiology in the South Pacific Region.

Reviewer #2: THIS IS AN IMPORTANT STUDY THAT NOT ONLY SERVES THE LOCAL POPULATION IN TERMS OF INDIVIDUAL AS WELL AS PUBLIC HEALTH, I.E. DISEASE PREVENTION, IDENTIFICATION / DETECTION / AWARENESS AND POSSIBLE TREATMENT BUT ALSO THE TRAVELING POPULATION IN ALL THE FIELDS MENTIONNED. AS THIS IS THE FIRST STUDY ON CHIK PREVALENCE IN THIS REGION I STRONGLY SUPPORT THIS PUBLICATION AFTER MINOR REVISIONS ARE DONE
